# The neglected importance of high-endemic ESBL sites

**DOI:** 10.1093/jacamr/dlac114

**Published:** 2022-11-03

**Authors:** Thor-Henrik Henriksen, Yitagesu Getachew, Ayelign Derebe Kindie, Behailu Tsegaye Mugoro, Soliyana Dejene Zewdie, Elias Tewabe, Wude Mihret, Selam Bogale Gissa

**Affiliations:** Department of Microbiology, Vestfold Hospital Trust, PO Box 2168, 3103 Tønsberg, Norway; Department of Internal Medicine, Yekatit 12 Hospital Medical College, Weatherall St, Addis Ababa, Ethiopia; Department of Internal Medicine, Yekatit 12 Hospital Medical College, Weatherall St, Addis Ababa, Ethiopia; Education Development Center Department, Yekatit 12 Hospital Medical College, Weatherall St, Addis Ababa, Ethiopia; Department of Microbiology, Yekatit 12 Hospital Medical College, Weatherall St, Addis Ababa, Ethiopia; Department of Internal Medicine, Yekatit 12 Hospital Medical College, Weatherall St, Addis Ababa, Ethiopia; Department of Internal Medicine, Yekatit 12 Hospital Medical College, Weatherall St, Addis Ababa, Ethiopia; Armauer Hansen Research Institute, Addis Ababa, Ethiopia; Department of Internal Medicine, Yekatit 12 Hospital Medical College, Weatherall St, Addis Ababa, Ethiopia

Recent reports in JAC-AMR on different aspects of antibiotic-resistant Enterobacterales, including the spread of ESBL, cover a number of issues that concern high-endemic settings, and need to be followed closely.^1–5^ A well-composed review and meta-analysis was a reminder about volume and rapidity of geographical spread of carriage of ESBL-carrying *Escherichia coli.*^5^ But, as also discussed by the authors, it is not known to what extent the increasing rates reflect better reporting from high-endemic areas rather than real growth in entire regions and globally. Rates of ESBL patterns and rapidity of spread vary enormously among nations, and to understand and halt this misfortune, high-endemic sites must be followed closely. Unfortunately, this is commonly where resources are low.

ESBL-related epidemiology is complicated, and can only be understood and managed globally when we follow important drivers, and understand how drivers depend on one another. This is of particular importance in high-endemic sites, where lack of information is worrying. As an example, the report on increased mortality and morbidity among patients with prolonged empirical antibiotic treatment in Addis Ababa, Ethiopia is by itself an important observation, but it also raises questions about the further context.^4^ Long-term empirical treatment with broad-spectrum antibiotics to hospitalized patients is exceptionally hazardous when the majority of individuals within the cohort are carriers even on admission; ESBL carriers should be protected against the selective pressure of antibiotics, certainly not preventively covered by broad-spectrum antibiotics.^6^ So, the remaining questions concern ESBL carriage rates in that area, and how carriage within the general population is acquired: through contaminated food, from animals, or from healthcare attendants. To manage spread of ESBL in that area, we need to know all of this.

Carbapenems are the recommended empirical treatment for bacterial infections when resistance to third-generation cephalosporins is a real threat.^3,7^ When >80% of Enterobacterales isolates causing healthcare-associated infection (HAI) are resistant to third-generation cephalosporins, criteria for empirical use of carbapenems are, in fact, fulfilled.^4^ Clinicians are thus trapped between the strong need to avoid carbapenems, and the lack of real-life alternatives. The cost of carbapenem usage in this setting has been a very rapid build-up of carbapenem resistance. This group of antibiotics has only been used in Ethiopia since 2013, and resistance to carbapenems became an important healthcare problem a few years later.^4^

Addis Ababa is certainly one of the many hotspots of ESBL-related antibiotic resistance worldwide.^4,8^ Avoidance of long-term empirical antibiotic treatment, and active tracing and removal of bacterial contaminants in healthcare starts with provision of bacteriological services and hospital infection prevention and control, including antimicrobial stewardship.^6,9,10^ This is where we are now, but the volume and quality of these services has only enabled exchange from empirical treatment to bacteriologically directed treatment for a small proportion of patients. Evidence-based management of bacterial diseases including ample access to bacteriological services, which is required to stop deterioration, is only possible if it becomes a strong healthcare priority.

We find it appropriate to end this commentary with the phrase, “It is essential that we remain vigilant about identifying ESBL-carrying Enterobacterales both in patient isolates and through surveillance studies”.^1^ Comprehensive surveillance studies, and establishment of sentinel functions in high-endemic areas, would be a task for international partnerships and the global community as a whole. We need to study how high-endemic patterns of ESBL are spread and burden community and healthcare, and also how shared bacterial lineages occur as aetiological agents, faecal carriage and environmental contamination inside and outside healthcare.^2^ Longitudinal studies of all these variables and their probable mutual dependence in high-endemic settings would help us to understand the situation and design feasible plans for better management.
